# Seasonal Pacing - Match Importance Affects Activity in Professional Soccer

**DOI:** 10.1371/journal.pone.0157127

**Published:** 2016-06-09

**Authors:** Daniel Link, Michael F. de Lorenzo

**Affiliations:** 1 Department of Exercise Science and Sport Informatics, Technical University of Munich, Munich, Germany; 2 School of Mathematical and Geospatial Sciences, RMIT University, Melbourne, Australia; Research Center for Sports Sciences, Health and Human Development (CIDESD), University of Trás-os-Montes e Alto Douro, Vila Real, Portugal, PORTUGAL

## Abstract

This research explores the influence of match importance on player activity in professional soccer. Therefore, we used an observational approach and analyzed 1,211 matches of German Bundesliga and 2^nd^ Bundesliga. The importance measurement employed is based on *post season consequences* of teams involved in a match. This means, if a match result could potentially influence the final rank, and this rank would lead to different consequences for a team, such as qualification for Champions League opposed to qualification for Europe League, then this match is classified as important; otherwise not. Activity was quantified by TOTAL DISTANCE COVERED, SPRINTS, FAST RUNS, DUELS, FOULS and ATTEMPTS. Running parameters were recorded using a semi-automatic optical tracking system, while technical variables were collected by professional data loggers. Based on our importance classification, low important matches occurred at the beginning of round 29. A two-way ANOVA indicates significantly increased FAST RUNS (+4%, d = 0.3), DUELS (+16%, d = 1.0) and FOULS (+36%, d = 1.2) in important matches compared to low important ones. For FAST RUNS and FOULS, this effect only exists in Bundesliga. A comparison of the two leagues show that TOTAL DISTANCE COVERED (+3%, d = 0.9), SPRINTS (+25%, d = 1.4) and FAST RUNS (+15%, d = 1.4) are higher compared to 2^nd^ Bundesliga, whilst FOULS is less in Bundesliga (-7%, d = 0.3). No difference in player activity was found between matches at the beginning of a season (round 1–6) and at the end of a season (round 29–34). We conclude that match importance influences player activity in German professional soccer. The most reasonable explanation is a conscious or unconscious pacing strategy, motivated by preserving abilities or preventing injury. Since this tendency mainly exists in Bundesliga, this may suggest that more skilled players show a higher awareness for the need of pacing.

## Introduction

Physical activity in soccer is subject to fluctuation. Research demonstrates that the total running distance declines after the most intensive five-minute interval [1], and also from the first 15 minutes of the first half compared to the second half [2]. Other studies found a decline of high-intensity running from the first to the second half [3]. Carling et al [4] found that early suspensions during a match lead to an increase of total distance covered, but no change in high intensive running and technical parameters. Harper et al ⁠[5] report a lower passing and tackling ratio in the extra time of a match.

The reasons for variations are highly complex. One explanation for decreased running activity in the second half or after high intensity periods could be fatigue, which can be caused by physiological factors such as reduced level of creatine phosphate [6] or exhausted muscle glycogen stores [7]. Others suggest that player activity is influenced by tactical considerations, such as the score line or the introduction of substitutes [8]. Alternatively, a reduced running performance can be attributed to conscious or subconscious pacing strategies that intend to preserve the ability to undertake high intensity activities when they are necessary [9–11].

Variations in performance do not only exist during single matches but also throughout a typical season [12–14], and can be influenced by team formation, team quality, opponent strength, location or temperature [15–19]. Another important factor—which is probably a key motivation for pacing—is the importance of a match. Particular matches at the end of a season may be more or less important to a team with respect to a reachable final rank.

Up to the knowledge of the authors, the only study which includes this factor was published by Bradley and Noakes [8]. The authors could not find any effect of match importance on total distance covered, but they reported a significant drop in high intensity running in the second half of critical matches. This decline was also found by Carling and Dupont [2], but without including the match importance factor, so it is unclear if the drop can be explained by the criticality of a contest. However, the study is limited to a small sample size of 55 players and details of the importance measure are vague, with the studying focusing on alternative factors.

The aim of this study is to assess the importance of matches throughout two seasons of Germany’s professional soccer leagues to study player activity with regard to match importance and league (skill level). For the evaluation we used physical (running distance, high intensive runs) and technical indicators (fouls, duels, attempts), from which we believe could be sensitive to pacing. We hypothesized that the activities in less important matches would decline, since players try to control their physical effort by reducing running, as well as their risk of injuries or suspensions by playing less aggressive in competitions for possession of the ball.

## Materials and Methods

### Subjects

In line with the objectives of our study, we apply a non-participative observational approach. The sample comprises 1,222 matches of Germans professional soccer leagues, Bundesliga and 2^nd^ Bundesliga, during the 2011/12 and 2012/13 seasons. Activity data was collected for players that were on the field for at least 45 minutes. The data is publicly available on the official website of the Bundesliga (www.bundesliga.de) so the usual approval from the research ethics committee was not required.

### Activity Parameters

For each match, player activity parameters were collected according to Table 1. Running parameters were recorded in 25 Hz using a semi-automatic optical tracking system (VISTRACK, by Impire Corp., Germany). Technical data was observed by professional data loggers based on video recordings. All data was collected by behalf of the German Professional Soccer League (DFL). The reliability of manual data collection was secured by DFL. Complete definitions, derivation of thresholds, validation procedures and results can be found here [20]. The validity and reliability of the tracking system have been described by Siegle, Stevens and Lames [21].

**Table 1:** Variables and operationalization. Variables represent data for one team in one match.

ID	Definition
TDC	Average distance covered (in m) by all field players. Game stoppages were included.
SPRINTS	Number of sprints. A sprint is a time period in which players speed is (1) higher than 4.0 m/s for at least 2 seconds, and (2), within these 2 seconds, reaches more than 6.3 m/s for at least 1 second.
FAST RUNS	Number of fast runs. A fast run is a time period that fulfils the criteria (1) of a sprint but not the criteria (2) but speed reaches more than 5.0 m/s for at least 1 second.
DUELS	Number of duels. A game action is called a duel if two player of different teams are in competition for the ball. A duel is always count for both teams.
FOULS	Number of fouls. A foul is a situation, where the referee interrupted the match cause of rough play; hand ball is not included.
ATTEMPTS	Number of attempts to score a goal. We do not differentiate between shots on target or shots next to or above the goal.
LEAGUE	*Bundesliga*
	*2*^*nd*^ *Bundesliga*
PERIOD	*Round 1–6*
	*Round 29–36*
IMPORTANCE	*Important (I)*: miPSC (match, team) >.05 and miPSC (match, opponent) > .05
	*Medium important (M)*, *subtype important (M-I)*: miPSC (match, team) >.05 and miPSC (match, opponent) < .05*)*
	*Medium important (M)*, *subtype low important (M-L)*: miPSC (match, team) < .05 and miPSC (match, opponent) > .05
	*Low important (L)*: miPSC (match, team) < .05 and miPSC (match, opponent) < .05

### Match Importance

To classify matches for teams as being important or less important, we apply a further development of an existing measure of match importance. The original measure, conceived by Anthony and Schembri [22], is based on a definition described in studies by Morris [23] and Schilling [24], where match importance is defined as the difference between the probability of a team achieving a season outcome given they win their next match; minus the probability of the team achieving the same outcome given they lose their next match. Therefore, our approach bases on the following concepts:

- *PSC*: post season consequence (PSC) is a set of positions that lead to equal consequences for a team after the season. As Table 2 shows, in the 2012/13 Bundesliga season there were 7 of these groups (e.g. PCS C = Bundesliga Champion, PCS CL = Champions League, PCS CLQ = Champions League Qualification, etc.). These may vary from season to season.
- *pPSC X (r*, *t)*: probability for a team t of finishing the season in PSC X in round *r*. This is calculated by using a cumulative binomial distribution function, which takes the points of t reached before round r and the estimated points necessary to reach a position in PSC X as an input. For example, a team on position 5 after round 30 might have a probability of *pPSC C(30*, *t)* = .02 to become Bundesliga champion and a probability of *pPSC CL (30*, *t)* = .23 to reach Champions League. Details of the method can be found here [22].
- *iPSC X (m*, *t)*: importance of a match *m* for a team *t* reaching PSC X. This is calculated by the difference between pPSC X after this round, given they win their next match; minus the pPSC X given they lose their next match. For example, a team on position 3 after round 31 might have a chance of becoming Bundesliga champion of *pPSCC* = .02 when they lose the next match, and of *pPSC C* = .22 if the win the next match. The importance of the match for t for becoming Bundesliga champion is i*PSC C (m*, *t)* = .20.
- *miPSC (m*, *t)*: maximum of all iPSC X of a match *m* for a team *t*.

**Table 2:** Ranking, post season consequences (PSC), schedule and match importance in Bundesliga 2012/13. There are nine PSC groups (C = Bundesliga Champion, CL = Champions League, CLQ = Champions League Qualification, EL = Europa League, ELQ1 = Europa League Qualification Round 1, ELQ1 = Europa League Qualification Round 2, M = Midfield RP = Relegation Play Off, R = Relegation). Opponent position shows the current ranking of a team’s opponent after round 33. The miPSC columns indicate, if the value is greater than 0.05 for the team (T) and its opponent (O). IMPORTANCE shows the classification of the matches in in important (I), medium important, subtype important (M-I), medium important, subtype low important (M-L) and low important matches (L).

Ranking after round 33					Schedule in round 34			
Pos	Team	Points	Goaldiff	PSC	miPSC T > 0.05	miPSC O > 0.05	Opponent position	IMPORTANCE
**1**	FC Bayern München	88	+79	C	0	1	8	M-L
**2**	Borussia Dortmund	66	+40	CL	0	1	17	M-L
**3**	Bayer Leverkusen	62	+25	CL	0	1	7	M-L
**4**	FC Schalke 04	52	+7	CLQ	1	1	5	I
**5**	SC Freiburg	51	+6	EL	1	1	4	I
**6**	Eintracht Frankfurt	50	+3	ELQ1	1	0	10	M-I
**7**	Hamburger SV	48	-10	ELQ2	1	0	3	M-I
**8**	Borussia Mönchengladbach	47	-3	M	1	0	1	M-I
**9**	Hannover 96	42	-5	M	0	0	15	L
**10**	VfL Wolfsburg	42	-5	M	0	1	6	M-L
**11**	VfB Stuttgart	42	-18	M	0	0	12	L
**12**	1. FSV Mainz 05	41	-2	M	0	0	11	L
**13**	1. FC Nürnberg	41	-9	M	0	0	14	L
**14**	SV Werder Bremen	34	-15	M	0	0	13	L
**15**	Fortuna Düsseldorf	30	-15	M	0	0	9	L
**16**	FC Augsburg	30	-20	RP	1	0	18	M-I
**17**	1899 Hoffenheim	28	-26	R	1	0	2	M-I
**18**	SpVgg Greuther Fürth	21	-32	R	0	1	16	M-L

The miPSC metric is used to define groups of IMPORTANCE (Table 1). A match *m* with teams A and B involved, is called *important for team A*, if *miPSC* (*m*, A) > .05 and *miPSC* (*m*, *B*) > .05. In other words, if winning or losing this match effects the probability of achieving a different the end-of-season consequence by more than 5% for both teams, then the match is important. In *low important* matches, *miPSC* do not exceed 0.05 for each of the teams, which means that there is no realistic chance to achieve a better or worse position. If *miPSC* is greater than 0.5 for only one team, than the match is classified as *medium important*. Here we also use two subgroups: If *miPSC* is greater than 0.5 only for team A, then this match as classified as medium important, subtype I for Team A, and as medium important, subtype L for Team B.

An example showing the match importance is given in Table 2. The match between FC Schalke 04 and SC Freiburg is classified as Important, because a win may secure each team a place in the Champions League. It can also be seen that the match 1. FC Nürnberg against SV Werder Bremen is Low important. This is because their standings and the standing of both teams has already been mathematically assured. The match Borussia Dortmund against 1899 Hoffenheim is Medium important. Although the match cannot affect the standing of Dortmund (subtype L), Hoffenheim could climb to position 16 if they win and simultaneously FC Augsburg would lose against SpVgg Greuther Fürth, which means that they will qualify for relegation play offs (subtype I).

### Statistical Analysis

The statistical analysis uses the data of one match and one team as a statistical unit. For our analysis, we group these units according to the conditions IMPORTANCE, LEAGUE and PERIOD (Table 1). The data is presented as the mean ± standard deviation. Before using parametric statistical test procedures, the assumptions of normality were verified. A two-way (2x3 design) analysis of variance (ANOVA) was tested on each dependent variable to examine the effect of IMPORTANCE and LEAGUE condition on player activity. Also, a one-way ANOVA was used to test the effect of PERIOD on activity. Differences of activity indicators between groups were determined by pair-wise Bonferroni post hoc analysis. Effect size was calculated according to Cohen by between group means divided by the standard deviation of both groups. Tests were conducted using 95% confidence (alpha level of 0.05). To control for type I error, a Bonferroni adjustment was applied by dividing the alpha level by the number of dependent variables. All statistical analyses were conducted using SPSS Statistics 22 for Windows (by IBM Corp., USA).

## Results

**Fig 1:**
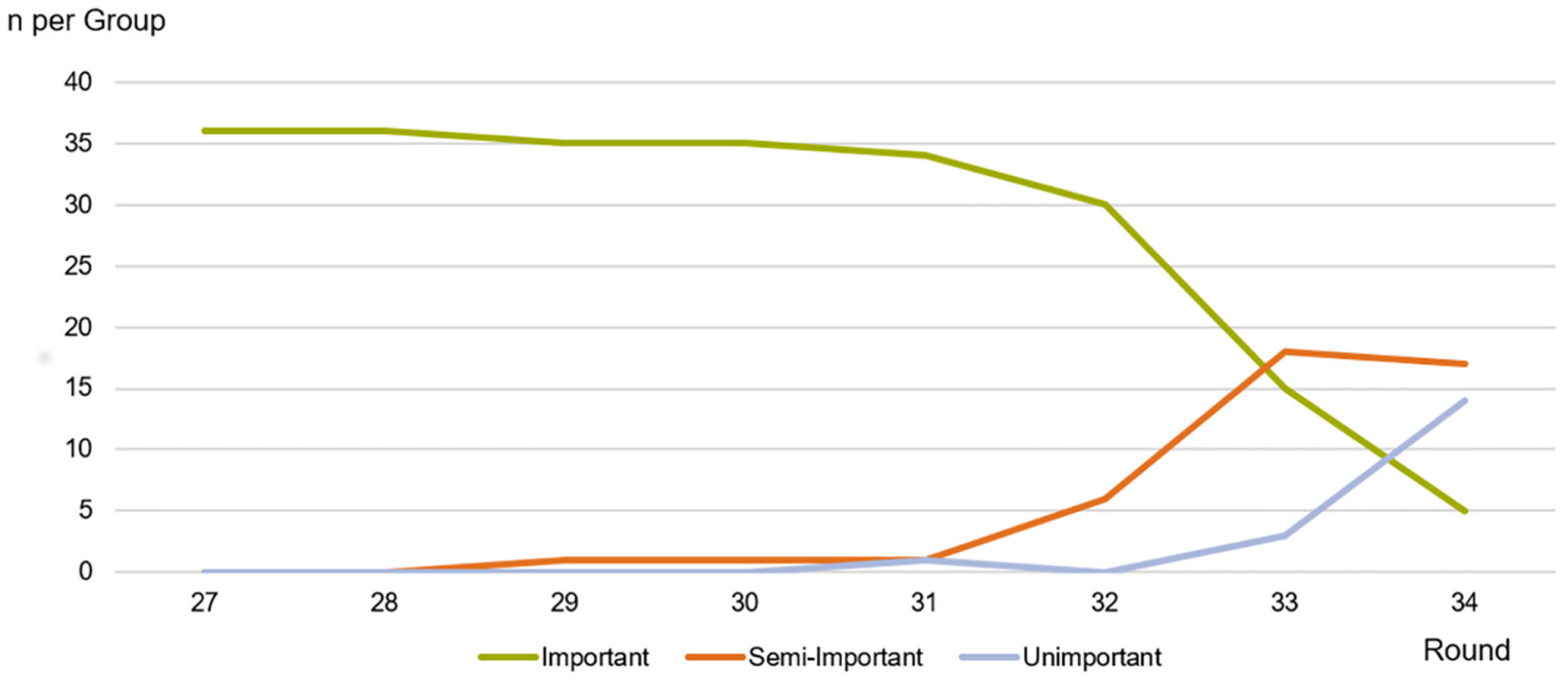
Group sizes of important, medium important and low important matches during the course of a season.

Our results include data from 1,222 matches. Due to poor data quality, e.g. heavy fog on the pitch, 11 matches were omitted, resulting in a final total of 1,211 matches. Based on our IMPORTANCE classification, medium important and low important matches occurred at the beginning of round 29. Up until the final round, the quantity of these matches increase due to the decrease of points that can be collected by teams (Fig 1).

Table 3 shows the results from the match statistics for important, medium important and low important matches. A two-way ANOVA indicates a significant main effect of IMPORTANCE on FAST RUNS, DUELS and FOULS. In important matches, the number of FAST RUNS and DUELS were higher compared to medium important (FAST RUNS: +6%, d = 0.5; DUELS: +9%, d = 0.6) and low important matches (FAST RUNS: +4%, d = 0.3; DUELS: +16%, d = 0.9). FOULS in important matches were only significantly increased compared to low important matches (+36%, d = 0.9). No differences were found for TDC, SPRINTS and ATTEMPTS. Also no differences were observed between the subtypes of medium important matches.

**Table 3:** Activity indicators for important, medium important and low important matches (left) and the subtypes of medium important matches (right).

	Important	Medium important	Low important	F_(2,2421)_	p <	Medium important (I)	Medium important (L)	F_(1,43)_	p <
**N**	2298	88	36			44	44		
**TDC**	113.9 ± 4.9	112.3 ± 4.4	113.0 ± 4.8	1.8	.19	112.8 ± 5.4	112.3 ± 4.7	0.1	.69
**SPRINTS**	146.3 ± 27.4	146.8 ± 28.0	149.0 ± 30.2	0.1	.91	145.7 ± 27.2	148.0 ± 34.3	0.1	.75
**FAST RUNS**	362.7 ± 36.1 ^23^	345.4 ± 31.5	349.9 ± 40.8	3.6	.028*	347.5 ± 47.6	343.4 ± 46.0	0,4	.69
**DUELS**	231.2 ± 33.2 ^23^	211.2 ± 34.5	198.9 ± 35.7	16.0	.001*	113.9 ± 19.2	114.3 ± 17.0	0.1	.92
**FOULS**	15.5 ± 4.3 ^3^	19.0 ± 4.6	11.4 ± 5.1	6.2	.002*	14.6 ± 4.9	15.9 ± 4.6	1.5	.23
**ATTEMPTS**	13.0 ± 4.8	13.6 ± 4.9	13.0 ± 4.7	0.7	.52	13.8 ± 4.9	13.3 ± 4.9	0.3	.71

*significant differences between groups (α < .05).

Table 4 shows the results differentiated between skill levels of teams. A significant main effect of LEAGUE was found for TDC (F_(1, 2421)_ = 28.5, p < .001), SPRINTS (F_(1, 2421)_ = 62.2, p < .001), FAST RUNS (F_(1, 2421)_ = 46.7, p < .001), and FOULS (F_(1, 2421)_ = 11.0, p *<* .001). In Bundesliga, TDC (+3%, d = 0.9), SPRINTS (+25%, d = 1.4) and FAST RUNS (+15%, d = 1.4) are higher compared to 2^nd^ Bundesliga, whilst FOULS is less in Bundesliga (-7%, d = 0.2). No significant differences were found for DUELS and ATTEMPTS.

**Table 4:** Activity indicators in important, medium important and low important matches grouped by league.

	Bundesliga						2^nd^ Bundesliga					
	All	Important	Medium important	Low important	F_(2,1205)_	p <	All	Important	Medium important	Low important	F_(2,1215)_	P <
**N**	1206	1144	48	14			1216	1154	40	22		
**TDC**	115.1 ± 4.3	116.1 ± 4.2	114.4 ± 3.9	116.0 ± 4.6	1.8	.18	111.3 ± 4.4	111.7 ± 4.5	110.4 ± 4.1	111.1 ± 4.0	0.9	.42
**SPRINTS**	161.8 ± 22.1	161.8 ± 21.3	165.3 ± 15.2	167.8 ± 27.5	0.6	.57	129.4 ± 24.1	130.9 ± 24.0	124.8 ± 23.5	137.0 ± 26.3	1,0	.38
**FAST RUNS**	385.1 ± 37.2	390.5 ± 36.1 ^2^	367.0 ± 31.5	375.3 ± 40.8	5.4	.006*	333.7 ± 37.8	335.2 ± 37.5	319.5 ± 40.0	333.7 ± 36.2	1.6	.19
**DUELS**	229.5 ± 34,5	230.6 ± 33,8 ^23^	206.7 ± 33,4	205.9 ± 38,1	11.1	.002*	230.7 ± 32.1	231.9 ± 32.5 ^3^	216.7 ± 28.0	205.9 ± 34.2	5.6	.005*
**FOULS**	15.0 ± 4.3	15.4 ± 4.4 ^3^	13.8 ± 4.9	11.3 ± 5.3	7.3	.001*	16.0 ± 5.9	16.4 ± 5.5	15.9 ± 6.0	15.0 ± 2.4	1.1	.36
**ATTEMPTS**	13.1 ± 5.0	13.0 ± 4.8	13.8 ± 4.7	12.5 ± 6.2	1.2	.3	13.2 ± 4.6	13.1 ± 5.2	13.2 ± 5.2	13.5 ± 6.2	0.1	.89

*significant differences between groups (α < .05),

^n^ indicates significant differences (post hoc analysis) to group n (^2^ Medium important, ^3^ Low important).

Significant LEAGUE × IMPORTANCE interactions were found for involvement with FAST RUNS (F_(2,2421)_ = 3.1, p< = .05) and FOULS (F_(2,2421)_ = 4.8, p < .05). In Bundesliga, FAST RUNS increased from medium important to important matches (+6%, d = 0.6). No significant change of this parameter was found between the importance groups in 2^nd^ Bundesliga. Also, in Bundesliga we observed more FOULS in important matches compared to low important matches (+36%, d = 0.9). In 2^nd^ Bundesliga, this effect did not exist. No significant interactions were found for the other dependent variables.

Table 5 shows the activity data for the groups of matches in the beginning and in the end of a season. No differences in player activity were found between these groups.

**Table 5:** Activity indicators in matches grouped by PERIOD.

	Round 1–6	Round 29–34	F_(1,858)_	p <
**N**	428	432		
**TDC**	110.8 ± 4.5	109.4 ± 4.6	0.1	.81
**SPRINTS**	146.1 ± 27.3	146.9 ± 29.2	0.1	.88
**FAST RUNS**	363,6 ± 32.5	362.1 ± 33.8	0,1	.91
**DUELS**	235.2 ± 34.9	233.2 ± 33.4	0.2	.78
**FOULS**	16.0 ± 4.4	15.6 ± 4.8	1,7	.19
**ATTEMPTS**	13.0 ± 4.8	13.2 ± 4.9	0.4	.53

## Discussion

The aim of this study was to identify influences of match importance on performance indicators in two seasons of German Professional soccer. In this context, the study employs a probabilistic model of match importance and applies the resulting classification on an analysis of activity.

Our classification method bases on the probability of a match to affect post-season consequences. With this, only the last games of the season are possibly classified as unimportant. Whilst this is one method, other techniques may also use different metrics. For example, matches against teams close in the table or below should be “must-win” games; and matches against opponents that are higher up in the table might be considered as less important, since they are very likely to lose anyway. This means, also in earlier stages of the season, before everything is decided, unimportant matches due to this criterion could occur. On the other hand, matches might be important even when they have no consequences on the final rank, e.g. when local rivals are playing against each other. One could also argue that players should be motivated to win every game; be it for the fans or teammates, for their own honor, or for the match bonuses. However, that may be, the applied importance quantity should provide a valid model for one central aspect of match importance.

To describe high intensity activities, we used the concept of fast runs and sprints. By using the number of these events instead of distances or time spent in speed intervals, we want to accent the acceleration component of running activity. We believe this is more promising to study pacing effects, since this factor is important for describing the energy costs in soccer [25,26].

The statistical analysis showed a medium effect of IMPORTANCE on DUELS in both leagues. This suggests that players are being more aggressive and play with a higher engagement due to the match having more meaning to it. IMPORTANCE also affects FAST RUNS and FOULS, but only in Bundesliga. This may suggest that more skilled players show a higher awareness for the need of pacing. This finding is consistent with [27] which report that high level marathon runners show more adaptions to the environmental temperature compared to mid-level runners.

In order to explain the different findings for SPRINTS (not increased) and FAST RUNS (increased), we qualitatively analyzed around 50 of these events based on video recordings. In our sample sprints are often committed in situations, when there is a fast counter attack, or the chance for an own scoring opportunity. These situations require maximum effort regardless if the match is important or not. In contrast to this, we observed that fast runs often occur in situations, where a player has to keep the team formation or move himself into a better position to receive a pass. However, in low important games there might be a tendency of players to not invest effort on these types of runs, as the consequences for the match are more indirect compared to the situations where they have to sprint.

TDC and IMPORTANCE are relatively independent. This is reasonable since TDC and intensive running are quite independent of each other [28]. With regard to ATTEMPTS, one could argue that importance affects this parameter, since player might reduce their effort in attacking or defending. Whatsoever, ATTEMPTS are not influenced by IMPORTANCE in our study—maybe because the effects do not exist or cancel each other. Another possible effect might be a higher activity of a team, for which a medium important match is more important—but this was not observed in our data. We believe that influence of match importance is too small to create an effect in the small sample here. Also, it might be that the intensity is more influenced by the result of the negotiated process between the two teams during the match than by considerations before the match. This is supported by findings reporting a high correlation of running parameters of both teams involved in a match [17].

No difference in player activity was found between matches at the beginning in the end of a season. This confirms that the decline at the end of the season in low important matches is not primary caused by fatigue. However, this is somehow remarkable since the accumulation of fatigue is a well-known phenomenon in soccer [29]. The data suggests that professional soccer teams are able to compensate individual fatigue up to a certain extent, e.g. by effective recovering procedures or by changing the starting line up from match to match. However this may be, there are also some other factors that could have affected match activity in our study like a reduced training load [30], or environment conditions [19].

Analysis of the condition LEAGUE finds more FOULS in 2^nd^ Bundesliga. One explanation is that players might have less technical skills, e.g. a lower passing accuracy [31], which can lead to situations where fouls are more likely. Also, TDC, SPRINTS and FAST RUNS in 2^nd^ Bundesliga are reduced compared to Bundesliga. This stands in contrast to the findings in UKs soccer published by Di Salvo et al [3], who reported the opposite effect in Premier League and Championship League. We think that there are two possible explanations. Firstly, playing style in the UK and Germany is different and this influences the relationships between the first and the second division in these countries. Secondly, this could be an artefact of the different tracking systems used. Up the experience of the authors, some systems are more sensitive for the location of the cameras than others. Since the stands are less high in the second leagues, this might increase the number of tracking lost which lead to less running distance measured due to interpolation. But this is somewhat speculative, with additional studies required to spread light on this finding.

## Conclusion

This study shows that professional soccer players significantly decline their match activity in low important matches at the end of the season. This effect seems primary not to be related to fatigue, but to a conscious or unconscious pacing strategy. Since this phenomenon mainly exists in Bundesliga, our data suggests that more skilled players show a higher awareness for the need of pacing. Although this may not be attractive to supporters, this strategy could be reasonable. This might help to preserve their abilities for finals or cup competitions, whilst also preventing injury or being sent off.
